# Advancing the use of real world evidence in health technology assessment: insights from a multi-stakeholder workshop

**DOI:** 10.3389/fphar.2023.1289365

**Published:** 2024-01-12

**Authors:** Ravinder Claire, Jamie Elvidge, Shahid Hanif, Hannah Goovaerts, Peter R. Rijnbeek, Páll Jónsson, Karen Facey, Dalia Dawoud

**Affiliations:** ^1^ National Institute for Health and Care Excellence, Manchester, United Kingdom; ^2^ GetReal Institute, Utrecht, Netherlands; ^3^ Pfizer nv/sa, Brussels, Belgium; ^4^ Erasmus University Medical Center, Rotterdam, Netherlands; ^5^ University of Edinburgh, Member Scottish Health Technologies Group Council, Edinburgh, United Kingdom; ^6^ National Institute for Health and Care Excellence, London, United Kingdom; ^7^ Clinical Pharmacy Department, Faculty of Pharmacy, Cairo University, Cairo, Egypt

**Keywords:** health technology assessment, regulatory, real world evidence, real world data, common data model, federated data network

## Abstract

**Introduction:** Real-world evidence (RWE) in health technology assessment (HTA) holds significant potential for informing healthcare decision-making. A multistakeholder workshop was organised by the European Health Data and Evidence Network (EHDEN) and the GetReal Institute to explore the status, challenges, and opportunities in incorporating RWE into HTA, with a focus on learning from regulatory initiatives such as the European Medicines Agency (EMA) Data Analysis and Real World Interrogation Network (DARWIN EU^®^).

**Methods:** The workshop gathered key stakeholders from regulatory agencies, HTA organizations, academia, and industry for three panel discussions on RWE and HTA integration. Insights and recommendations were collected through panel discussions and audience polls. The workshop outcomes were reviewed by authors to identify key themes, challenges, and recommendations.

**Results:** The workshop discussions revealed several important findings relating to the use of RWE in HTA. Compared with regulatory processes, its adoption in HTA to date has been slow. Barriers include limited trust in RWE, data quality concerns, and uncertainty about best practices. Facilitators include multidisciplinary training, educational initiatives, and stakeholder collaboration, which could be facilitated by initiatives like EHDEN and the GetReal Institute. Demonstrating the impact of “driver projects” could promote RWE adoption in HTA.

**Conclusion:** To enhance the integration of RWE in HTA, it is crucial to address known barriers through comprehensive training, stakeholder collaboration, and impactful exemplar research projects. By upskilling users and beneficiaries of RWE and those that generate it, promoting collaboration, and conducting “driver projects,” can strengthen the HTA evidence base for more informed healthcare decisions.

## 1 Introduction

Health technology assessment (HTA) is a multidisciplinary process that assesses the value of health technologies to inform decision-making, aiming to enhance equity, efficiency, and quality in healthcare systems (O’Rourke et al., 2020). It is widely used throughout Europe to make decisions about the reimbursement and pricing of healthcare technologies, including new medicines. Estimates of relative effectiveness, healthcare use and costs are key inputs for assessing effectiveness, cost-effectiveness, and budget impact, which are required for HTA recommendations in several countries. Companies and HTA organisations face multiple challenges in obtaining and generating such evidence in support of their products.

Traditional HTA approaches primarily rely on randomised controlled trials (RCTs) to generate clinical evidence. However, there is growing recognition of the importance of integrating real-world evidence (RWE) derived from real-world data (RWD) sources into HTA processes. RWE may provide a more comprehensive understanding of interventions’ effectiveness and safety in clinical settings, and address some of the evidence gaps faced by companies and HTA organisations. However, the uptake of RWE for HTA has been slow compared with regulatory decision making.

The European Medicines Agency (EMA) has established the Coordination Centre for the Data Analysis and Real World Interrogation Network (DARWIN EU^®^) (darwin-eu.org) (EMA, 2021). It aims to provide access to valid and trustworthy RWE from across Europe on diseases, populations and the use and performance of medicines. This will increasingly support regulatory decision-making, which is often followed by HTA to support reimbursement decisions (EMA, 2023).

To explore the current landscape and prospects of incorporating RWE in HTA, a multi-stakeholder workshop titled “Advancing Real-World Evidence in Health Technology Assessment” was convened by the Innovative Medicines Initiative (IMI) funded European Health Data and Evidence Network (EHDEN) project (ehden.eu) (IMI, 2018), in collaboration with the GetReal Institute. EHDEN aims to enable large-scale analysis of health data in Europe by building a large federated data network of standardised data (EHDEN, 2018). Part of the project involves supporting the transition towards outcomes-driven healthcare systems in Europe, by adopting the use of a federated data network approach for HTA purposes. The GetReal Institute is an independent, member-led non-profit organisation emerging from two IMI projects with the mission to facilitate the adoption and implementation of RWE in regulatory and HTA decision-making in Europe. The aim was to foster collaboration, share experiences, and identify key strategies to facilitate the use of RWE in HTA. This article presents an overview of the workshop discussions, highlighting key findings, recommendations, and areas for future development.

## 2 Materials and methods

The workshop was convened with relevant stakeholders to discuss the current state, challenges, and future directions of integrating RWE into HTA processes. Experts and stakeholders were selected based on their expertise and experience in RWE and HTA. Key individuals from academia, regulatory agencies, HTA organisations, industry, and patient organisations were invited to ensure a diverse range of perspectives.

The workshop was designed as a half-day event, comprising three panels focused on specific topics related to RWE and HTA integration. Each panel consisted of a presentation followed by a moderated discussion. The first panel discussed the progress and future of DARWIN EU^®^, and reflections from HTA organisations on plans for adoption of RWE. The second panel focused on reflections from industry, patient organisations, and academics. The final panel discussed the potential of EHDEN and GetReal Institute in supporting RWE integration in HTA.

Following the panel presentations, open discussions were held among the workshop participants. These discussions allowed for the exchange of ideas, identification of common challenges, and exploration of strategies to overcome barriers hindering the wider adoption of RWE in HTA. The participants shared their perspectives, experiences, and recommendations based on their respective domains of expertise. In addition, two audience polls were conducted to gather insights and perspectives from the attendees. The first poll aimed to identify the areas within HTA where RWE could help resolve decision-critical evidence gaps. The second poll aimed to determine the areas where initiatives like EHDEN and the GetReal Institute could provide support for HTA. Both polls enabled participants to select more than one option to accurately capture their views.

The data collected during the workshop, including audience poll results, presentation materials, and discussion notes, were compiled. Key themes, common challenges, and potential recommendations were identified and synthesised to provide a comprehensive understanding of the workshop outcomes.

## 3 Results

### 3.1 Panel 1: EMA, DARWIN EU^®^ & reflections from HTA organisations

The first panel focused on the establishment of the DARWIN EU^®^ and its Coordination Centre by EMA. The discussions highlighted the ambitious goal of providing access to valid and trustworthy RWE from across Europe, encompassing diseases, populations, and the use and performance of medicines. The panel highlighted the value of standardising health data using the Observational Medical Outcomes Partnership Common Data Model (OMOP CDM) maintained by the Observational Health Data Sciences and Informatics (OHDSI) community (www.ohdsi.org) which could help to realise the necessary need to scale up real-world data studies across Europe (Hripcsak et al., 2015).

The second part of the panel focused on reflections from HTA organisations and their plans for adopting RWE. The panel highlighted that there is growing interest in utilising RWE for HTA decision-making. The need for improved trust in RWE and availability of good quality data were identified as key factors limiting its adoption. HTA organisations expressed the need for data that reflect the target population and regional variations in healthcare. The panel recognised the potential benefits of RWE in speeding up access to new treatments and reducing the cost of drug development programs. Some HTA organisations are investing in the development of frameworks and best practices for planning, conducting, and reporting RWE studies, though there is scope for cross-border collaboration in such efforts. The panel emphasised the importance of upskilling technical staff and committees to evaluate the quality and appropriateness of RWE.

The moderated panel discussion examined the fundamental differences between regulators and HTA organisations regarding RWE use cases. Regulators focus on safety and efficacy, while HTA organisations consider relative clinical effectiveness and cost effectiveness. However, there are potential overlapping use cases that could benefit both regulators and HTA organisations, such as characterising a given disease population and its natural history. This understanding of shared goals can shape the choice of data partners for DARWIN EU^®^ and EHDEN. The selection of these data partners is driven by stakeholders’ specific questions and the need to generate relevant evidence.

The panel acknowledged the increasing interest and adoption of the OMOP CDM in Europe, particularly stimulated by the EHDEN project and the recent DARWIN-EU^®^ initiative. This is also resulting in the establishment of so called national nodes that drive the adoption of the data model and its use in collaborative studies at the national level (www.ohdsi-europe.org).

### 3.2 Panel 2: reflections from stakeholders

The second panel of the workshop featured reflections from relevant HTA stakeholders representing industry, patient organisations, a health data medical research funder, and a multi-stakeholder initiative focused on RWE generation for healthcare decisions.

The industry panelist highlighted the potential value of RWE in informing reimbursement decisions but identified challenges such as data standardisation and collaboration. The patient representative expressed support for RWE but emphasised the need for resources and training for patients to understand and engage with it. The medical research funder representative emphasised infrastructure and real-time data and the RWE initiative representative highlighted challenges in data quality and the lack of expertise in utilising RWD.

The moderated panel discussion addressed the need for training and upskilling staff, particularly in healthcare decision-making bodies. Efforts to develop educational materials and align various organisations and initiatives were discussed. The potential to extend learnings from COVID-19 projects to other conditions was explored, emphasising the importance of identifying impactful “driver projects”. Aligning EU member states on RWD requirements and involving decision-making bodies in data infrastructure discussions were identified as crucial steps. Accounting for real-world context in RWE studies and involving data custodians to ensure appropriate data utilisation were also highlighted.

### 3.3 Panel 3: RWE and HTA: how can EHDEN and GetReal Institute help?

The final panel of the workshop focused on the role of EHDEN and the GetReal Institute in supporting the generation and use of RWE in HTA. The GetReal Institute, from its previous work with the GetReal Think Tank, identified three focus areas of interest to stakeholders; reducing barriers to using secondary data sources, bridging the gap between RCTs and RWE, and addressing evidence needs of healthcare decision-makers.

HTA use cases using EHDEN were discussed, including examples in chronic obstructive pulmonary disease (Kent et al., 2021), cancer, and COVID-19. The use cases demonstrated how EHDEN’s real-world data can be used in economic models, provide insights into cancer survival, and assess treatment effectiveness for COVID-19.

**FIGURE 1:**
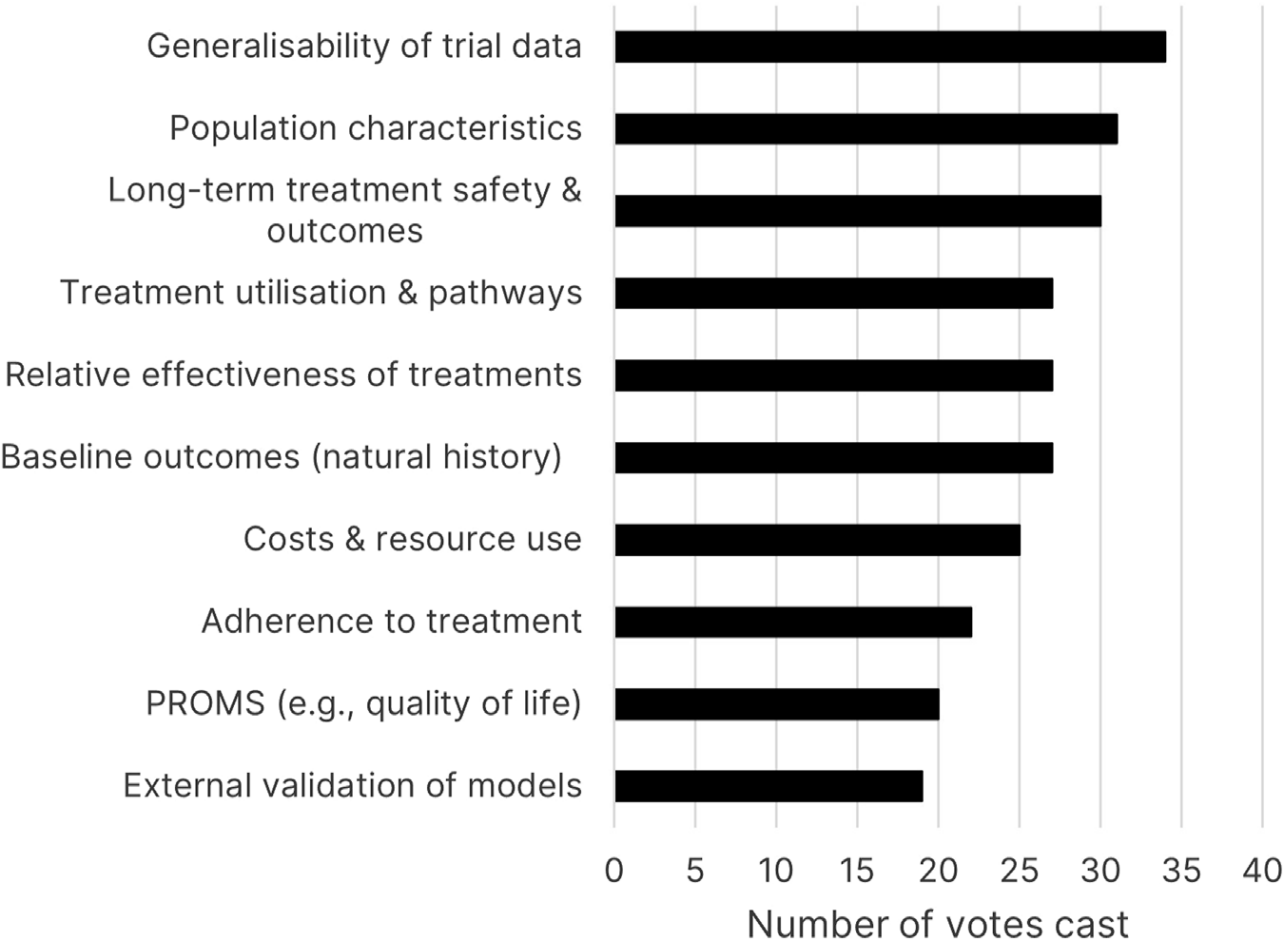
Poll results for “Where does HTA experience decision-critical evidence gaps RWE could help to resolve?” (PROMS = patient-reported outcome measures).

Two audience polls were conducted during the moderated panel discussion. The first asked, “Where does HTA experience decision-critical evidence gaps that RWE could help to resolve?.” “Generalisability of trial data” was the most selected option in this poll (Figure 1), though several other gaps received a high number of votes, such as disease population characteristics, long-term health outcomes, and identifying treatment pathways. Notably, quantifying relative effectiveness received many votes, despite HTA organisations traditionally highly prioritising randomised evidence for this purpose. These results indicate that HTA processes grapple with multiple issues that suitable RWE may help to inform.

**FIGURE 2:**
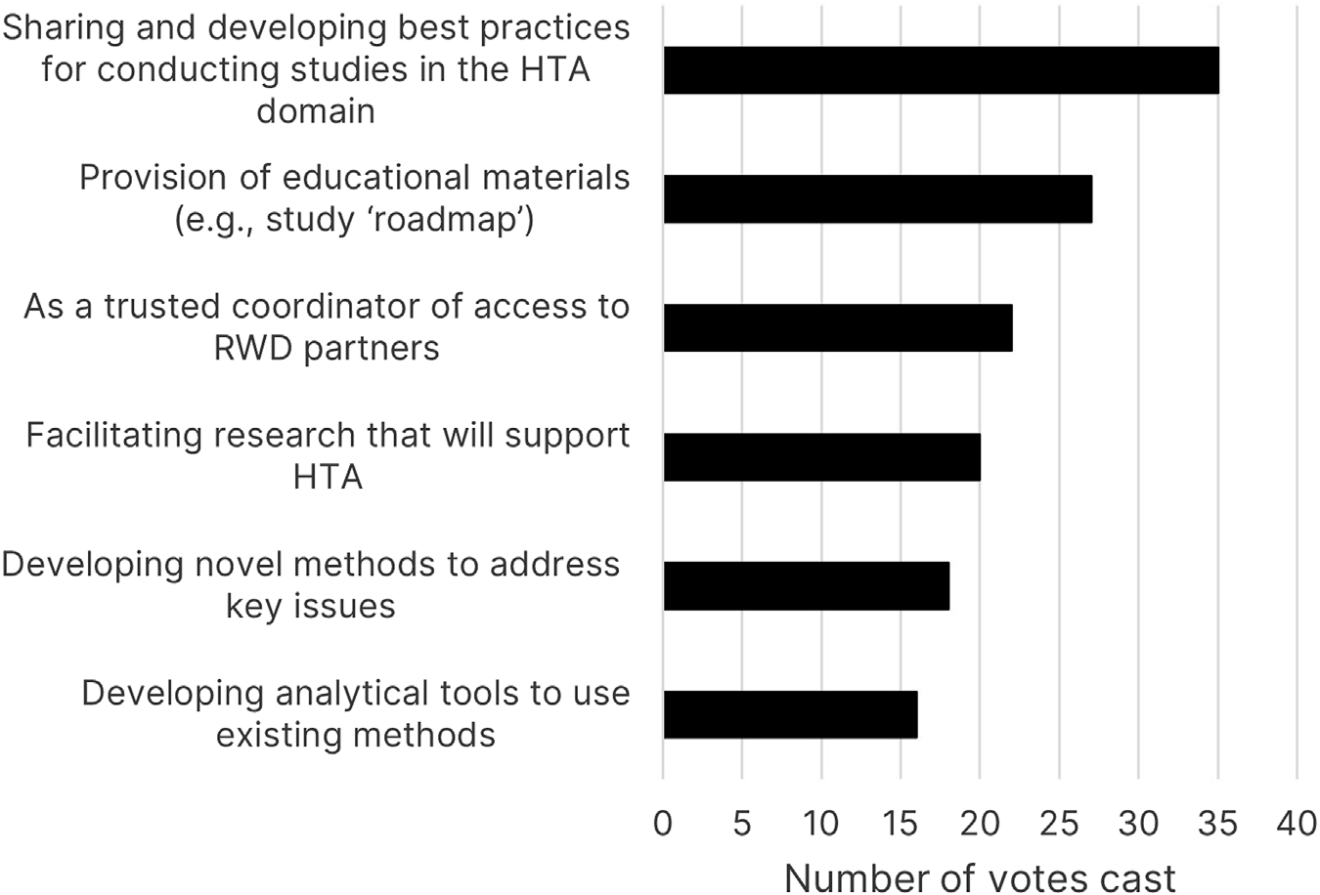
Poll results for “Where should initiatives like EHDEN and GetReal Institute focus their support for HTA?”

The second poll asked, “Where should initiatives like EHDEN and GetReal Institute focus their support for HTA?”. “Sharing and developing best practices for conducting studies in the HTA domain” was the most selected option (Figure 2), with “provision of educational materials” the second most popular answer.

Training and upskilling in RWD was a key theme in the moderated panel discussion. It was recognised that a multidisciplinary approach is needed. This should encompass wide-ranging learning materials including topics such as phenotyping, study design, and analytical approaches. These resources should be accessible across a variety of training levels (undergraduate through to postgraduate degrees), and to HTA staff and relevant stakeholders, such as industry. Educating healthcare workers responsible for data collection is also essential, and it should be demonstrated how the collected data informs their practice and contributes to meaningful outcomes. Both EHDEN and the GetReal Institute have educational platforms targeted to a broad audience through the EHDEN Academy (academy.ehden.eu) and the GetReal Academy (getreal-academy.org). Example courses that are directly relevant to the integration of RWE in HTA include “Real-World Evidence in Medicine Development” on the GetReal Academy, and the “Health Technology Assessment” course on the EHDEN Academy.

Discussions also revolved around the future and next steps for RWD adoption in HTA. Engaging stakeholders in ongoing discussions and projects was emphasised to drive impactful advancements in HTA. The importance of “driver projects” was emphasised, as they provide practical experience and learning opportunities. It was noted that more of these projects are needed, and prompt action is necessary due to the rapid pace of change. Involving key stakeholders in the conduct of driver projects, and structured organisation of projects were considered vital for success.

## 4 Discussion

The workshop provided valuable insights into the integration of RWE in HTA and identified key challenges and opportunities in this domain. There is a clear divergence between the acceptability of RWE for regulatory decision-making and for HTA decision-making. Traditional HTA approaches favour randomised evidence to support assessments of clinical and cost effectiveness, but HTA organisations will increasingly be presented with healthcare technologies with regulatory approval underpinned by RWE. Limited trust in RWE, concerns about data quality, and a limited understanding of what best practice is when it comes to RWE, were identified as barriers to wider adoption in HTA.

Generally, the findings are in line with published literature on barriers to adoption and use of RWE in HTA (Hogervorst et al., 2022). To address the barriers to RWE adoption, there is a need for comprehensive and multidisciplinary training and education initiatives. Starting from undergraduate levels, extending to healthcare providers responsible for data collection, and healthcare payers who make decisions about reimbursement, these efforts should aim to enhance awareness, knowledge, and expertise in assessing the quality and appropriateness of RWE.

The success of RWE integration in HTA depends on collaboration and engagement among stakeholders (Facey et al., 2020). Initiatives like EHDEN and the GetReal Institute play a crucial role in facilitating coordination, providing neutral forums, and developing resources to promote best practices and recommendations. To build trust and demonstrate the value of RWE, the identification and execution of impactful “driver projects” is essential. Initially, these projects should focus on characterising patient populations and the natural history of diseases, as these are comparatively simple analyses that can provide tangible, useful evidence for HTA quickly.

Further driver projects that focus on other identified evidence gaps, such as examining the generalisability of RCT evidence—which may require more complex studies—would be valuable. Where possible, driver projects should address evidence gaps that are common to the HTA and regulatory spaces. Such projects have been initiated within EHDEN focusing on key challenging areas for HTA including extrapolation of cancer survival data beyond the time horizon of clinical trials and assessing relative effectiveness of treatments (Claire et al., 2022). These are two key challenging methodological areas for the use of RWE in HTA, and future driver projects should similarly aim to address evidence gaps.

Based on the workshop discussions, the following recommendations are proposed to advance the integration of RWE in HTA:

• Develop and Promote Training Resources: A comprehensive strategy should be developed to create, develop, and promote training resources to upskill users and beneficiaries of RWE in HTA. These resources should cover various disciplines and target different levels of education, from undergraduates to established HTA professionals.
• Identify and Execute “Driver Projects”: Key driver projects that can have a substantial impact on methodology development and build trust in the use of RWE should be identified. These projects should focus on areas of high relevance to HTA and involve collaboration among stakeholders to ensure recognition and support for their outcomes.
• Start with “Easy-Win” Projects: Initiating projects that address the characterisation of patient populations and the natural history of diseases, particularly in areas of overlap with the regulatory space, is a good starting point. These easy-win projects provide tangible outcomes and pave the way for further advancements in RWE integration in HTA.
• Collaboration and Stakeholder Engagement: Continued and deeper collaboration among stakeholders, including HTA organisations, researchers, industry representatives, and patient organisations, is crucial for the successful integration of RWE in HTA. Efforts should be made to maintain engagement, foster discussions, and drive projects that align with the vision and development of RWE adoption in HTA.

The article summarises the key findings and recommendations derived from a multi-stakeholder workshop. The insights gained from this workshop have the potential to inform future strategies and initiatives aimed at promoting the use of RWE in HTA, to support evidence-informed and patient-centred healthcare decision-making and, ultimately, better health outcomes.

## Data Availability

The original contributions presented in the study are included in the article/Supplementary material, further inquiries can be directed to the corresponding author.
